# The Influence of Listening to Music on Adults with Left-behind Experience Revealed by EEG-based Connectivity

**DOI:** 10.1038/s41598-020-64381-x

**Published:** 2020-05-05

**Authors:** Yin Tian, Liang Ma, Wei Xu, Sifan Chen

**Affiliations:** 1 Bio-information College, ChongQing University of Posts and Telecommunications, ChongQing, 400065 China; 2 Sichuan Heguang Clinical Psychology Institute, ChengDu, 610074 China

**Keywords:** Cognitive neuroscience, Emotion

## Abstract

The human brain has a close relationship with music. Music-induced structural and functional brain changes have been demonstrated in the healthy adult. In the present study, adults with left-behind experience (ALB) were divided into two groups. The experimental group (ALB-E) took part in the music therapy experiment with three stages, including before listening to music (pre-stage), initially listening to music (mid-stage) and after listening to music (post-stage). The control group (ALB-C) did not participate in music therapy. Scalp resting-state EEGs of ALB were recorded during the three stages. We found no significant frequency change in the ALB-C group. In the ALB-E group, only the theta power spectrum was significantly different at all stages. The topographical distributions of the theta power spectrum represented change in trends from the frontal regions to the occipital regions. The result of Granger causal analysis (GCA), based on theta frequency, showed a stronger information flow from the middle frontal gyrus to the middle temporal gyrus (MFG → MTG) in the left hemisphere at the pre-stage compared to the post-stage. Additionally, the experimental group showed a weaker information flow from inferior gyrus to superior temporal gyrus (IFG → STG) in the right hemisphere at post-test stage compared to the ALB-C group. Our results demonstrate that listening to music can play a positive role on improving negative feelings for individuals with left behind experience.

## Introduction

Adults with Left-behind experience (ALB) generally refers to an individual that underwent an extended period of time in childhood without residing with parents and was instead brought up by grandparents or relatives. This is caused by unbalanced economic development within a geographical region and has become an emerging social phenomenon. This phenomenon is common in rural China, Philippines, Indonesia, and other countries^1^. After the period of lacking parents’ care and communication, ALB usually present strong psychological insecurity and mistrust for other people. In social occasions, ALB are not willing to take the initiative to establish good interpersonal relationships with others. From an early age, they will have a mentality of inferiority and cowardice which affects subsequent development. This can make ALB more likely to opt to escape and deal with problems in a negative way. In addition, ALB often show emotional indifference in their psychology. Long-term communication with parents is relatively sparse and there is little sense of family warmth, which will affect their overall personality and emotional attitude. This can result in a lack of social interest and methods for ALB, and social anxiety frequently occurs during their interactions with others. Previous studies have reported that people who have left-behind experience may present with depression, anxiety, loneliness, and lower satisfaction for life compared to those who have no left-behind experience^2^. Researchers conducted a survey of 4857 involving children and adults with left-behind experience in Chongqing Municipality of China, and found that the proportion of depression symptoms was up to 24.8%^3^. The left-behind experience may have a profound impact on an individual’s future development. In order to minimize the psychological trauma caused by the left-behind experience, music-assisted therapies, including singing, listening to music, and other music activities, have been used to improve the enthusiasm for life of left-behind children^4^.

Music-assisted therapy has been well evaluated in the study of cognitive disorders. Researchers studied the efficacy of music therapy in the treatment of depression among working-age people and found that compared to standard care alone, individual music therapy combined with standard care can be more effective for treatment of depression^5^. Previous studies explored the mechanism of music therapy in the treatment of depression and suggested that the increased effectiveness of music therapy may be because active music-making within the therapeutic frame offers the patient opportunities for new aesthetic, physical, and relational experiences^6^.

With the development of modern imaging technology, the influences of music on brain structure and function be greatly resolved. Based on children’s morphological research, long-term music training can shape the development of brain structures, including frontal, temporal, and parietal regions^7^. In a brain study of both adult musicians and non-musicians, a difference in the central anterior gyrus neural activity was observed^8^. These studies indicate that music could cause changes in brain plasticity.

Modern neuroimage research has shifted its focus from the localization of specialized neural activation to the interpretations in neural dynamics. Brain network analysis based on graph theory is an important tool to assess the interactions among multiple brain areas^9–11^. In contrast with functional magnetic resonance imaging (fMRI), EEG with a high time resolution has the ability to study oscillations within a dynamic human brain architecture^12^. However, conventional scalp recordings are inadequate to resolve EEG source locations, because scalp voltage contains a mixture of underlying source activity and volume conduction^9,13^. To resolve the problem, independent component analysis (ICA) decomposes resting-state EEG signals^14^. ICA can remove artifacts and concatenate individual-subject EEG epochs across subjects over time to apply the group data algorithm^14^. In addition, as a statistical methodology for time series inference, Granger causality analysis (GCA) uncovers dynamic interactions in a complex nervous system^15–18^. Since many measured electrophysiological signals were characterized by spectral properties, spectral GCA approaches have received considerable attention^19^.

In the present study, three stage group-ICA and GCA methods were used to explore the influence of listening to music on the dynamic effective connectivity of ALB. The groups were: before listening to music (pre-test), listening to music (middle-test) and after listening to music (post-test). Power spectrum analysis was initially performed to find characteristic frequency-bands. Group-ICA was then utilized to decompose resting-state EEG signals into a number of independent components (ICs) which served as time series. Weight minimum norm estimate (WMNE) was used to calculate cortical activities of these ICs which served as network nodes. Finally, based on characteristic frequency-bands obtained by power spectrum analysis, the spectral GCA was constructed to research changes of network connectivity. In this study we test the effects of listening to music in ALB by observing cortical activities with characteristic rhythm based on scalp EEG recording. We assumed that music therapy could alter on brain connectivity and improve negative feelings of ALB.

## Results

### Spectral analysis

**Figure 1:**
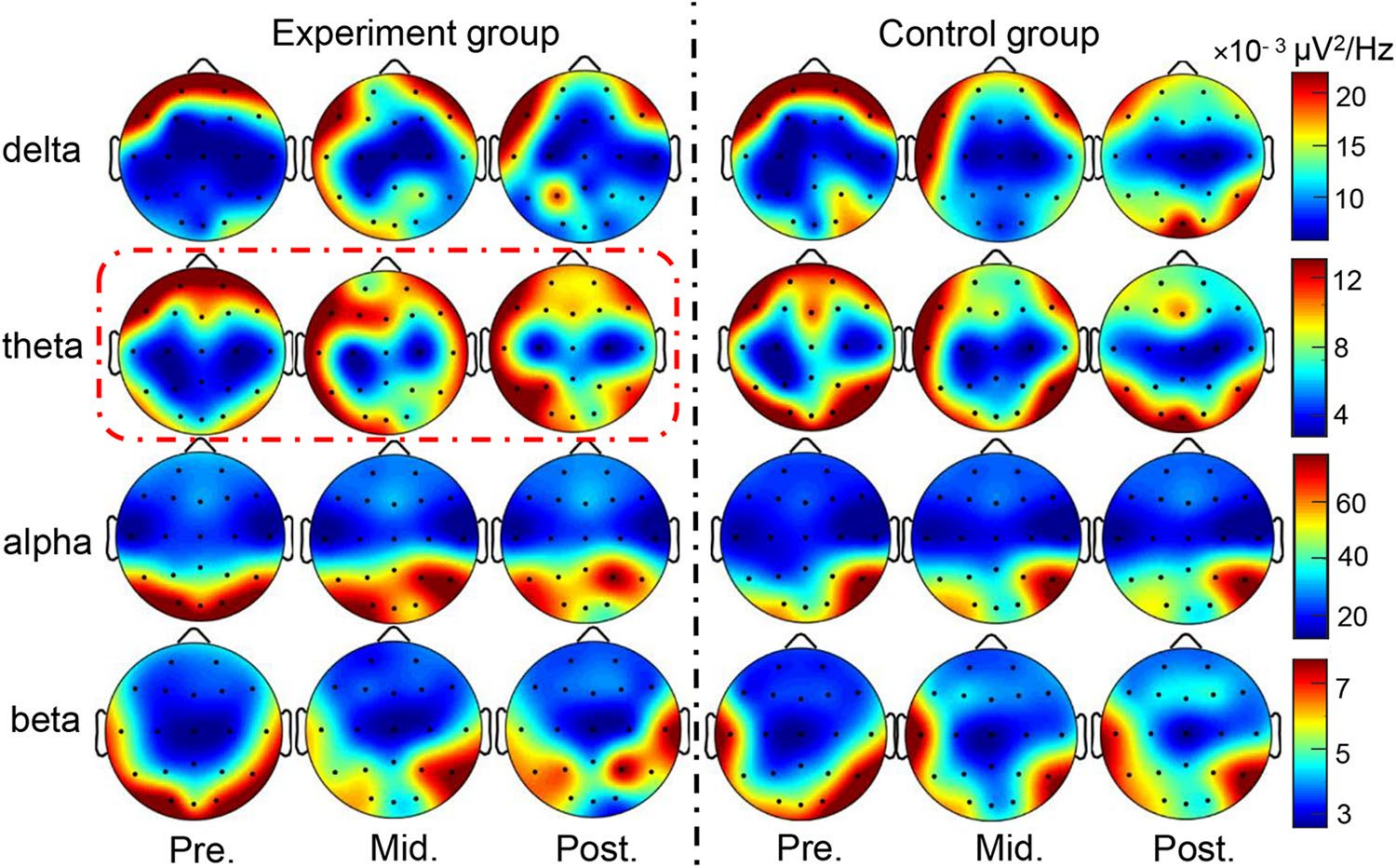
The average power map of the experimental group and the control group under four frequency bands. Pre.: pre-test stage; Mid.: mid-test stage; Post.: post-test stage. Red dotted box: significant difference on theta band between stages.

**Figure 2:**
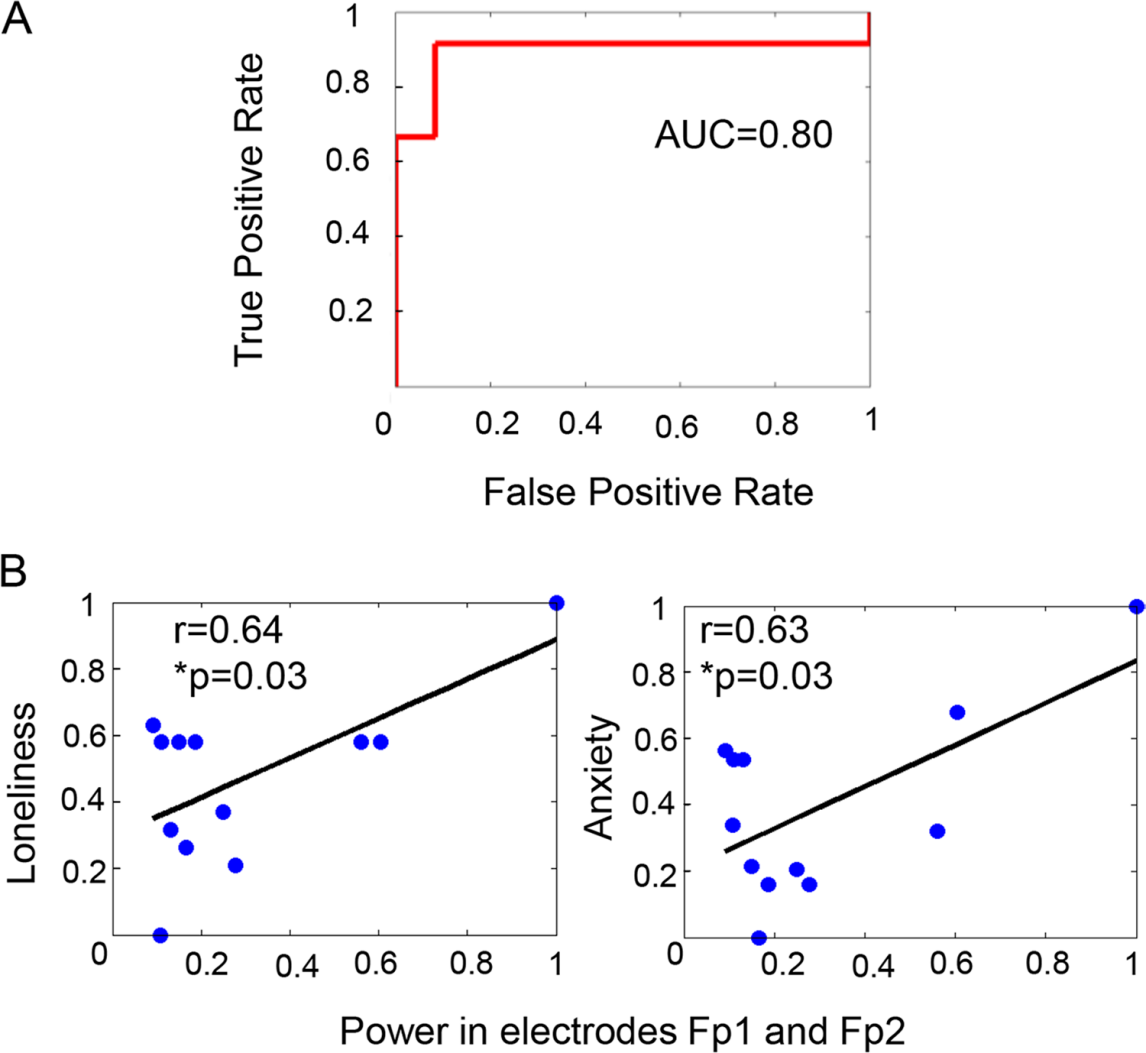
(**A**) ROC curve. (**B**) The scales analysis results at the pre-test stage of the experiment group under theta power. ROC: Receiver Operating Characteristic; AUC: Area Under Curve.

Significant differences were found in the theta frequency band (*p* < 0.05) of the experimental group, indicating the theta rhythm may be a characteristic frequency band of ALB in music-assisted therapy. In the experimental group, the distribution of the theta power spectrum had a trend of changing from frontal regions to occipital regions with the progress of listening to music (Fig. 1). Variances in extracted power spectrums of occipital regions (electrodes O1 and O2) and frontal regions (electrodes Fp1 and Fp2) between pre-stage and post-stage were analyzed. The ratio of the sum power spectrum at the occipital electrodes to that at the frontal electrodes served as a feature and support vector machine (SVM) with radial basis function (RBF) kernel and a classifier for the identification of theta power spectrum changes between pre-test and post-test. By leave-out cross validation (LOOCV), classification accuracy was 83.3% and the area under ROC curve was 0.80 (Fig. 2A), revealing that listening to music continuously over a long period of time can lead to theta energy transferring from the frontal to the occipital region. In addition, positive correlations between the sum power at the pre-frontal lobe electrodes and the scale scores of loneliness (*r* = 0.64, *p* = 0.03) and anxiety (*r* = 0.63, *p* = 0.03) were also observed, respectively (Fig. 2B). There was no significant difference for all frequency bands among three test stages for the control group (one-way ANOVA: all F < 1, p > 0.05).

### Network core node and resilience

**Figure 3:**
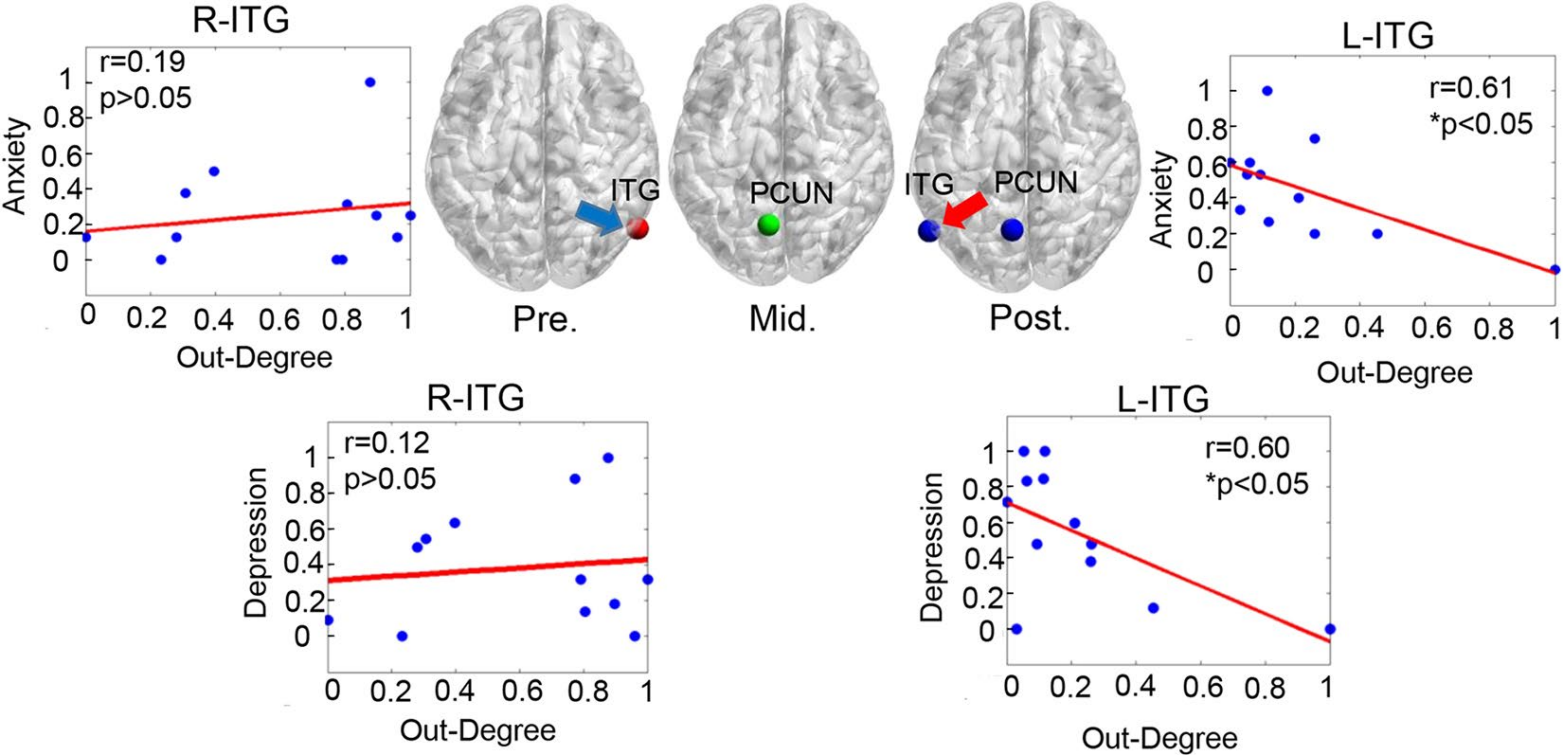
The changes of core nodes at three stages for the experiment group. ITG: inferior temporal gyrus, PCUN: precuneus. R-ITG: right-ITG, L-ITG: left-ITG.

A network consists of many nodes. Some of nodes play a critical role on modulating a large number of connections, which were called core nodes. In neuroscience, core nodes represent structurally or functionally important brain regions, and they are linked to different brain areas to facilitate functional integration^20^. Previous studies found that core nodes are easily affected by Alzheimer’s disease, psychosis, major depression, and other mental diseases^21^. In this study, we explored the effect of listening to music on core nodes of the causal brain network in ALB. For the experimental group, core nodes shifted to different locations during the different experimental stages (Fig. 3): the right inferior temporal region during the pre-test stage (Fig. 3, left); the precuneus during the mid-test stage (Fig. 3, middle); and the left inferior temporal region and the precuneus region during the post-stage (Fig. 3, right).With the progression of listening to music, core nodes transferred from the right hemisphere to the left hemisphere. Only during the post-test did the out-degrees of the left inferior temporal region (ITG) demonstrate significant negative correlations with the depression scores (*r* = 0.60, *p* = 0.04) and the anxiety scores (*r* = 0.61, *p* = 0.03). For the control group, the core nodes were located at the same location in the right inferior temporal region over the course of the experiment.

**Figure 4:**
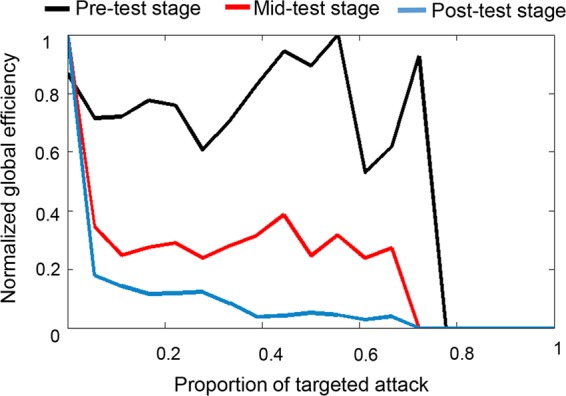
Mean global efficiency changes by targeted attacking over 12 subjects in brain networks of the experiment group.

The area of curve (AUC) was used to evaluate the network resilience^22^. For the experimental group, a significant main effect of test-stage was observed (*F* = 4.44, *p* < 0.05), and the post hoc t-test further demonstrated that the global efficiency at pre-test was larger than at post-test (all *p* > 0.05). Although there was no significant difference between mid-test and post-test, the global efficiency at post-test stage showed a decreased trend when compared to the mid-stage (Fig. 4). For the control group, no significant difference was found in network resilience between the three test stages.

### Granger causality

**Figure 5:**
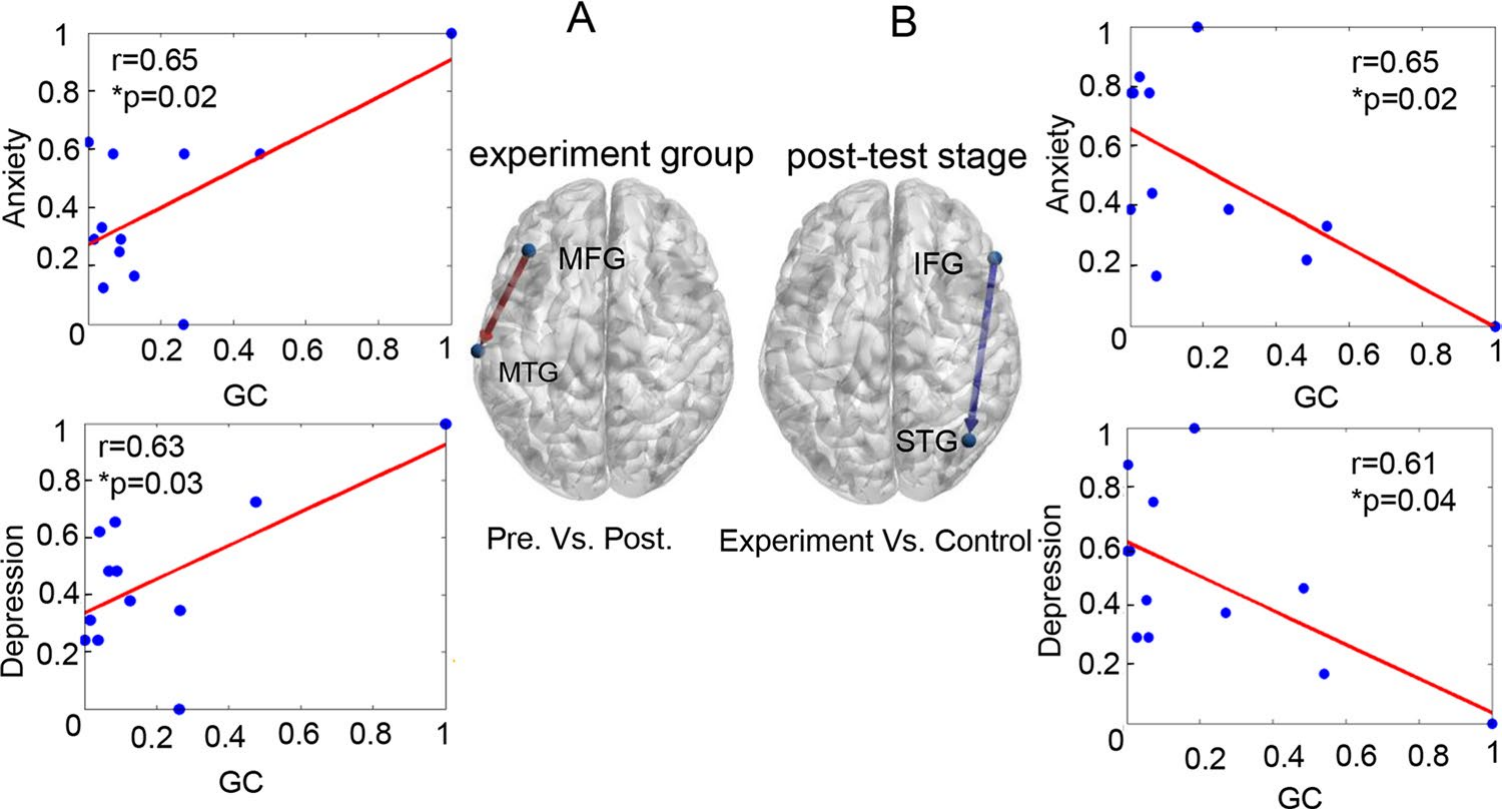
Significant differences of causal connectivity caused by listening to music. (**A**) Experimental group. Red arrow indicated that the GC value of MFG → MTG at the pre-test was stronger than the post-test stage. GC represented casual connectivity value at pre-test stage of the experiment group. (**B**) Post-test stage. Blue arrow indicates GC values of IFG to STG in the experimental group is weaker than that in the control group. GC represented casual connectivity value at post-test stage of the experiment group.

Granger causal (GC) information flows between cortical regions were calculated for different stages and displayed graphically with respect to the mean group z-score. For the experimental group, the GC value of the causal flow from the middle frontal gyrus (MFG) to the middle temporal gyrus (MTG) at the pre-test stage was significantly greater than GC at the post-test stage (*p* < 0.05, FDR correction; red arrow in Fig. 5A). Notably, both depression scores and anxiety scores were significantly positively correlated with the GC values of MFG to MTG at the pre-test stage (depression: *r* = 0.65, *p* = 0.02; anxiety: *r* = 0.63, *p* = 0.03; Fig. 5A). For the post-test stage, the GC values of the causal flow from the inferior frontal gyrus (IFG) to the superior temporal gyrus (STG) in the experimental group was significantly less than GC in the control group (*p* < 0.05, FDR correction; blue arrow in Fig. 5B). Both depression and anxiety scores were significantly negatively correlated with GC values of IFG to STG for the experimental group (depression: *r* = 0.61, *p* = 0.04; anxiety: *r* = 0.65, *p* = 0.02; Fig. 5B). For the control group, no significant correlations between depression and anxiety and the GA values were observed. In addition, we also checked the possible cognitive changes caused by listening to music from the point-of-view of the complex network parameters of the whole brain including node degree, clustering coefficient, local efficiency, and global efficiency; no significant effects were observed for these parameters.

## Discussion

Using ICA and GCA, the present study investigated the influence of music-assisted therapy on adults with left-behind experience. We found that the theta power spectrums and brain connectivity varied with listening to music. The findings demonstrated that music played a positive role on improving negative feelings of ALB.

### Characteristic band with theta rhythm

EEG theta rhythm was first reported by Walter and Dovey, who observed their occurrence in cases of sub-cortical tumors^23^. Since that time, a permanent bond between theta rhythm and cognition was established. Previous studies have shown that the EEG theta rhythm was especially sensitive to processing emotional stimuli^24^. Theta power at various scalp locations can be enhanced by affective content characterized by arousal and negative valence^25^. The theta rhythm also plays an important role in the emotional network of human brains^26^. Our previous findings reported theta effects on music tempo^10,11^. A large number of studies have shown that left-behind children have more emotional problems compared to non-left behind children^27^. The differences in power spectrums between the three test stages under theta rhythm observed here indicated that music-assisted therapy can the influence of the emotions of ALB.

The prefrontal cortex plays an important role in the regulation of emotion. Previous studies showed that an increase of prefrontal cortex activity could reflect the aggregation of negative emotion; few positive emotions were expressed^28^. The strong theta power in posterior regions such as occipital area represented motivated emotional attention processing (bottom-up processing). Our results showed that the theta power of frontal regions was positively correlated with loneliness and anxiety (negative emotions) for the experimental group prior to listening to music (Fig. 2B), indicating that the activation in the frontal regions may reflect the negative psychology of ALB in the pre-test stage (before listening to music). We did not find a significant relationship between scales and theta power change in the occipital regions. Theta energy was transferred from the frontal regions to the occipital regions with the continuation of listening to music (Fig. 1, theta), suggesting that the music-assisted therapy can promote the release of negative feelings in ALB.

### Changes of core nodes

Previous studies have shown that the brain cognitive impairment could lead to alteration of the core nodes^29^. In the present study, for both the experimental group and the control group, the core nodes were distributed in the parietal-temporal region, which was consistent with the distribution of the theta rhythm^30^. Among them, the bilateral inferior temporal gyri (ITG) were involved in emotional experience, and the precuneus was an important core region of healthy brain. Using fMRI, the functional network’s degree at the left temporal node was significantly negatively correlated with depression scales after treatment with antidepressant drugs^31^, suggesting that the increased degree at the left temporal lobe was associated with the improvement of the patient’s course of disease. Researchers also investigated the functional connectivity with depressive patients, and found that the functional connectivity of the left temporal lobe with depressed patients was significantly decreased compared to the healthy^32^. The previous findings suggest that the left temporal lobe plays an important role in the process of depression, and that the transfer of the core nodes from the right to the left temporal lobe may indicate a potential positive effect on ALB after listening to music in the present study.

Results of this study revealed that the brain network of adults with left-behind experience had greater resilience before listening to music than after listening to music (Fig. 4). These results were similar to a previous study where brain networks with major depressive disorders had greater resilience compared to a healthy control group^33^. Non-random networks (such as the brain network) have often showed more vulnerability to targeted attack because their degree distributions are more heterogeneous than random networks, and the removal of higher degree nodes would cause a more serious effect on the global integrity of the network^33,34^. This indicates a protective mechanism for avoidance of fast deterioration of ALB.

### Difference of GC connectivity

In the present study, no significant differences were observed on network characteristics. It could be that local differences were undetectable in relation to the average of the whole brain or that the time course of listening to music was too short (six weeks).

During emotional processing, the left and right hemispheres have a different division of labor in regulating the function of emotions. A common view is that the left and right hemispheres are related to the processing of positive^35^ and negative emotions^36^. Figure 5A showed a stronger information flow (red arrow) in the left hemisphere during pre-test compared to post-test; the GC values of MFG to MTG increased with the increased anxiety and depression scores during the pre-stage, which indicated that the strong top-down control from the MFG could inhibit positive emotion processing and bias towards feeling negative emotion in ALB. As shown in Fig. 5B the experimental group showed a weaker information flow than the control group in the right hemisphere during the post-test, and the GC values of IFG to STG increased with the decreased anxiety and depression sores. This suggests that the weaker top-down control from IFG could weaken negative emotions and improve negative effects with left behind experience of ALB.

## Conclusion

In this work, the brain changes of ALB were investigated based on the combined advantages of power spectrum analysis, ICA, source localization, and graph theory. The findings showed that theta rhythm played an important role on the effects induced by music in ALB, and revealed that brain network changes of core nodes and effective connectivity, indicating that music can improve people’s positive attitude towards life.

## Methods

### Participants

Twenty-four right-handed male subjects without any history of mental or neurological problems (age = 22 ± 3 (SD) years) were recruited for the experiment at the University of Electronic Science and Technology of China. All subjects had not previously learned music or played musical instruments and had at least one year of left-behind experience before they were 12 years old. Informed consent was signed prior to the study, and subjects also received a monetary compensation after concluding the experiments. All experiments were approved by the ethical committee of University of Electronic Science and Technology of China. All experimental methods were conducted in accordance with the ethical guidelines determined by the National Ministry of Health, Labour and Welfare and the Declaration of Helsinki (BMJ 1991; 302:1194).

### Experiment design and data recording

**Figure 6:**
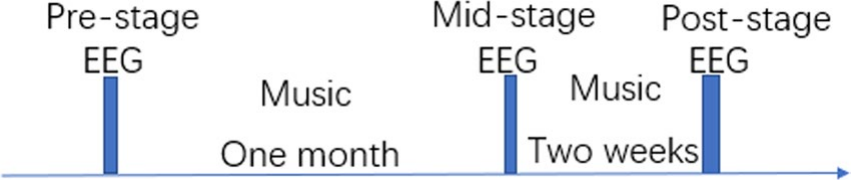
Time courses of resting-state EEG recordings and music therapies. Resting-state EEGs of both the control group and the experimental group were recorded three times, i.e. pre-stage, mid-stage, and post-stage. The control group did not perform music therapies.

Subjects were randomly divided into the experimental group or control group with 12 subjects in each group. The experimental group (ALB-E) took part in the music therapy experiment and the control group (ALB-C) did not perform music therapy. Before listening to music, the two-sample t-test was performed between the experimental group and the control group on the depression score and the anxiety score, separately. There were non-significantly difference [anxiety score: t = 0.18, p = 0.86) and depression score (t = 1.3, p = 0.21)]. Therapeutic music can provide emotional support, prompt users to experience feelings of success, reduce anxiety, rebuild value systems and behavior patterns, learn new interpersonal attitudes and a sense of responsibility in view of different problems. The selected music tracks in the present study could improve negative feelings such as anxiety, depression, irritability, hopelessness, loneliness, becoming easily tired, sense of vale, insomnia, uneasiness, or fear. The music tracks in the experiment and the targeted issues were shown Table 1. A 20-channel UEA-BZ EEG system was used to record EEG data and the sampling rate was set to 512 Hz. The experiment was divided into three stages: before listening to music (pre-test), where five minutes of the resting state data were recorded for both the experimental and control groups; subjects in the experimental group listened to music for 30 minutes each day (mid-test), where the music tracks in Table 1 were chosen according to their feelings. The control group did not perform in any treatment. After one month, five minutes of resting state data were recorded for both the experimental and control groups. The subjects in the experimental group listened to music for 30 minutes a day, while the control group did not listen to music. After two weeks, the resting-state data of 5 minutes for both experiment group and control group were recorded (post-test) (Fig. 6).

**Table 1:** The information of music used in the experiment.

*Music tracks*	*Music author*	*Target individual*
Turkey March	Mozart	Tired
Grand round dance	Chopin	Anxiety, depression
Pour Elise	Beethoven	Irritability, hopelessness
Dance of the Cygnets	Tchaikovsky	Lonely
Heaven and hell from Cancan	Offenbach	Lonely
Tales from the Vienna Woods	Johann Strauss	Sense of value
Moonlight	Beethoven	Irritability, insomnia
The night of the barren hills	Moussorgski	Uneasiness, fear

### EEG preprocessing

**Figure 7:**
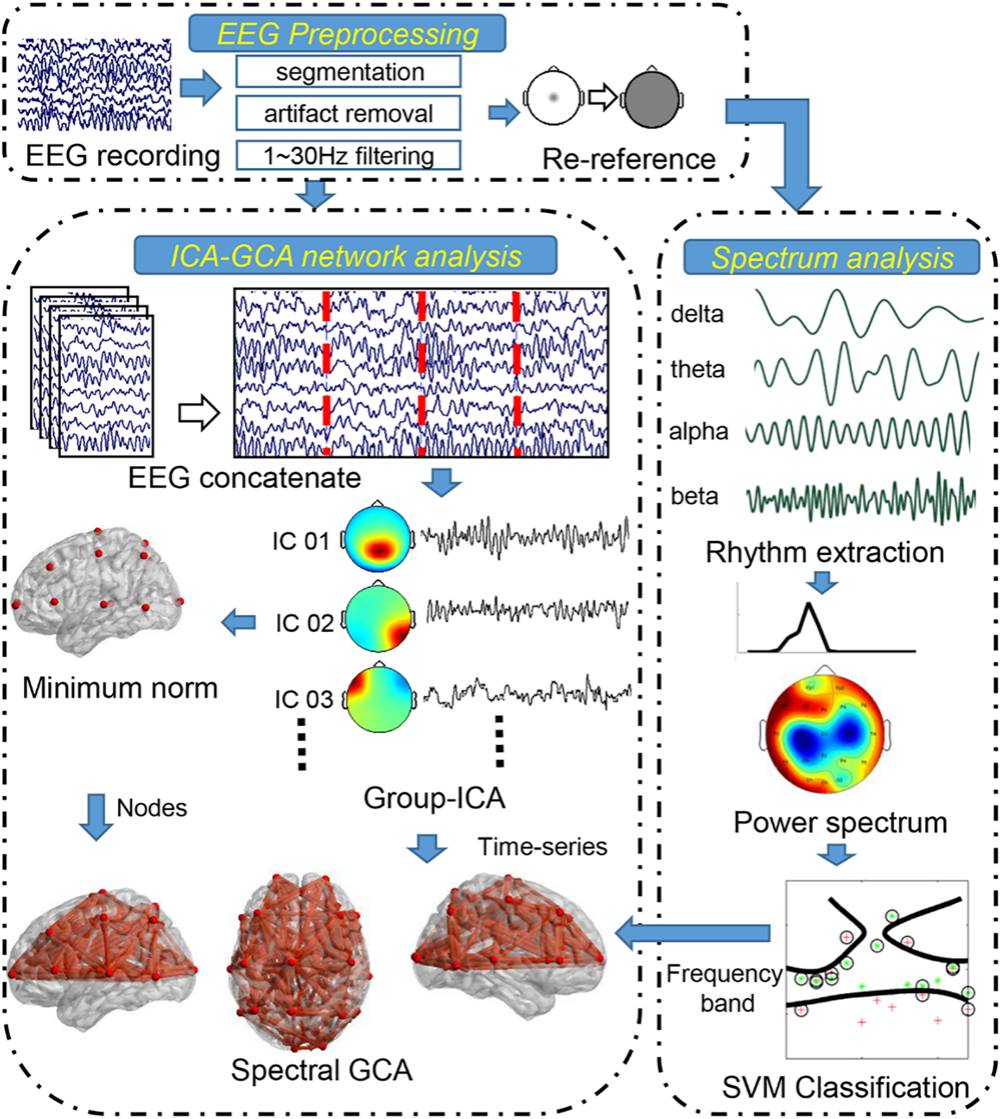
EEG data analysis processing.

The EEG recordings were divided into 4 s epochs. Epochs with blinks and eye movement were rejected offline and an artifact criterion of ± 60 μV was used at all other scalp sites to reject epochs with excessive electromyography (EMGs) or other noise transients. EEG recordings were filtered with a band-pass of 1–30 Hz. The data were then re-referenced by averaging reference^37,38^. A detailed data analysis process is shown in Fig. 7.

### Power spectral analysis

Delta (1~4 Hz), theta (4~8 Hz), alpha (8~13 Hz), and beta (13~30 Hz) rhythm were extracted by Fourier transformation^39^. The power spectrum of each rhythm signal was then calculated using the pwelch algorithm^40^. The average power spectrum of all data epochs for each subject was used as the final power spectrum for the subject. The average power of the signal on the rhythm was obtained by the averaging power of all the frequency points of the power spectrum. Finally, an ANOVA was performed to find characteristic frequency-bands which reflected the differences of EEG power between test stages induced by music-assisted therapy.

### Grouped independent component analysis

All subjects’ data segments were connected in series, and then ICA was carried out for group data. ICA can be expressed mathematically by Eq. 1 in Table 2.

**Table 2:** Formulas of independent component analysis (ICA) and Source location analysis.

Name	Formula	Remarks
Independent component analysis (ICA)	$$\begin{array}{c}X=A\ast S\,\,{\rm{E}}{\rm{q}}.\,1\end{array}$$	$X$: the EEG data of 20 channels after cascaded. $A$: the mixed matrix. $S$: the unknown independent component.
Source location analysis	$$\begin{array}{c}S=WX\,{\rm{E}}{\rm{q}}.\,2\end{array}$$$$\begin{array}{c}W=R{A}^{T}{(AR{A}^{T}+{\lambda }^{2}C)}^{-1}\,{\rm{E}}{\rm{q}}\,3\end{array}$$$$\begin{array}{c}\lambda =\frac{trace(AR{A}^{T})}{trace(C)\ast SN{R}^{2}}\,{\rm{E}}{\rm{q}}.\,4\end{array}$$	$$X$$: the mixed matrix. $$S$$: the corresponding cortical power. $$W$$: the linear inverse operator. $$C$$: the covariance matrices of the noise, $$R$$: the covariance matrices of the noise. $$A$$: the gain matrix. signal-to-noise ratio ($$SNR$$): A fixed value of 5, which reflected the value in the evoked response experiments^41^.

Source location analysis.

WMNE was performed on scalp maps of selected ICA components to find the maximal densities of their cortical sources^41,42^. For valid ICA components, the source location was estimated by Eq. 2–4 in Table 2.

**Figure 8:**
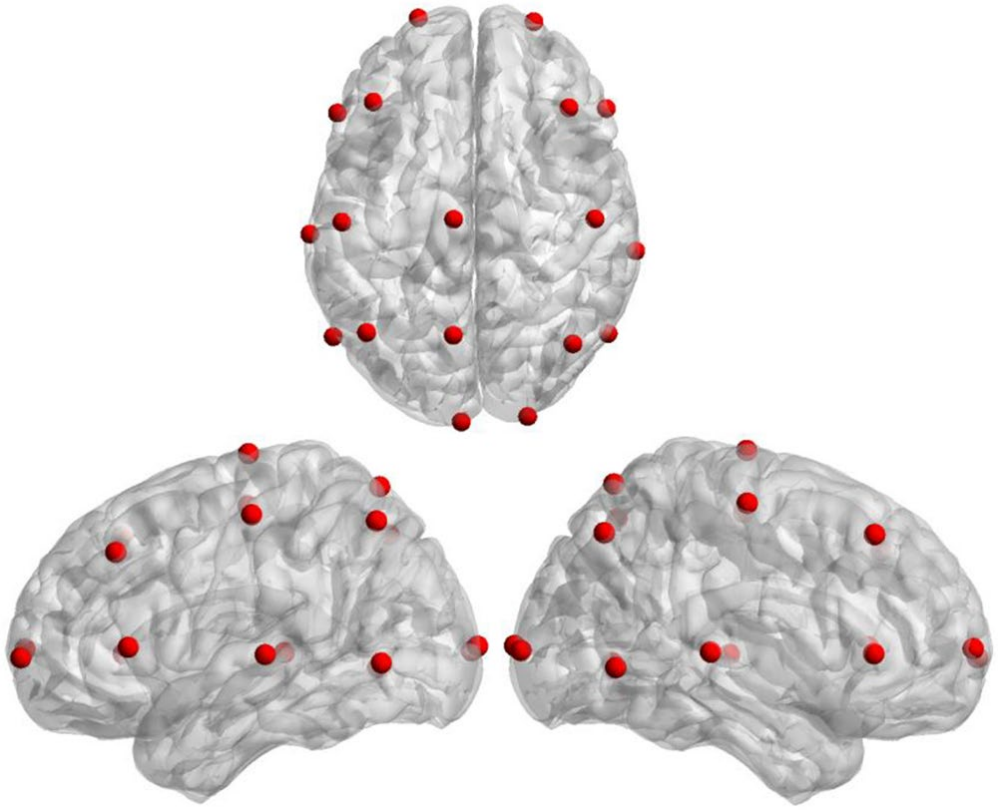
The cortical locations of 18 ICs. ICs: independent Components.

Here, a 3-shell realistic head model was adopted for EEG source activities estimation, where the conductivities for the cortex, skull, and scalp were 1.0 Ω^− 1^ m^− 1^, 1/80 Ω^− 1^ m^− 1^, and 1.0 Ω^− 1^ m^− 1^respectively. The solution space was restricted to the cortical grey matter, the hippocampus, and other possible source activity areas, consisting of 15002 cubic mesh voxels with 10 mm inter-distance. The lead field matrix was calculated by the boundary element method (BEM)^43^. The source locations of 18 independent Components (ICs) can be seen in Fig. 8.

### Spectral GC analysis (GCA)

For each time series, a multivariable autoregressive model was constructed and used to describe the dataset via Eq. 1 in Table 3. Then, in order to estimate coefficient matrix *A* and covariance matrix *C* of *E(t)*, we multiplied Eq. 1 from the right by ${X}^{T}(t-k)$, where k = 1,2,…,p. Taking expectations, the Yule-Walker equation was obtained by Eq. 2–3 in Table 3.

**Table 3:** Formulas of spectral GC analysis (GCA).

Name	Formula	Remarks
Spectral GC analysis (GCA)	$\mathop{\sum }\limits_{l=0}^{p}A(l)\ast X(t-l)=E(t)$ Eq. 1	$X$: the multichannel data vector. $A(l)$: the coefficient matrix, $A(0)$: the unit matrix. $E(t)$: unbiased white noise with zero mean and its noise covariance was defined as ${\rm{C}}$. $p$ was the model order which was decided by Akaike Information Criterion(AIC)^44^.
	$$\begin{array}{c}\mathop{\sum }\limits_{l=0}^{p}A(l)\ast R(-k+1)=0\end{array}$$ Eq. 2	$R(n)=E(X(t)\ast {X}^{T}(t+n))$, representing the covariance matrix when the $X(t)$ step was ${\rm{n}}$ and being able to be approximately calculated by the Eq. 3.
	$$\begin{array}{c}\mathop{R}\limits^{ \sim }(n)=\frac{1}{N-n}\mathop{\sum }\limits_{i=1}^{N-n}X(i){X}^{T}(i+n)\,{\rm{E}}{\rm{q}}.\,3\end{array}$$	$N$: the number of sample points.
	$$\begin{array}{c}H(f)={(\mathop{\sum }\limits_{j=0}^{p}A(j){e}^{-2\pi fj})}^{-1}\,{\rm{E}}{\rm{q}}.\,4\end{array}$$	$H(f)$: the transfer function in frequency domain.
	$$\begin{array}{c}S(f)=H(f)C{H}^{\ast }(f)\end{array}$$ Eq. 5	$S(f)$: spectral matrix in frequency domain. $\ast$: the complex conjugate transpose. $f$: the frequency band of interest.
	$$\begin{array}{c}{I}_{j\to i}(f)=-ln\left(1,-,\frac{(C(i,j)-{(C(j,j)}^{2}/C(j,j)))|{H}_{ij}(f){|}^{2}}{{S}_{ii}(f)}\right)\,{\rm{E}}{\rm{q}}.\,6\end{array}$$	${I}_{j\to i}(f)$: spectral causal connectivity from channel $j$ to channel $i$.

For multiple realizations, we computed the above quantity for each realization, and averaged across all the realizations to obtain the final estimate of the covariance matrix^45^.

Applying the Levinson-Wiggins-Robinson (LWR) algorithm to Eq. 1 and Eq. 2 in Table 3^46^, the value of *A* and *C* can be obtained. The transfer function *H(f)* in the frequency domain can then be computed by Eq. 4 in Table 3. The spectral matrix in the frequency domain was obtained by Eq. 5 in Table 3. The final spectral causal connectivity from channel j to channel i was represented by Eq. 6 in Table 3. For each subject, a permutation test was used to remove meaningless edges caused by noise. The number of permutations was set to 1000.

### Network core nodes and resilience

For each subject, betweenness centrality was used to define network core nodes and the threshold was set to the sum of mean and standard deviation of betweenness of all nodes^47^. For each group, the sign rank test was used to find statistical core nodes.

Network resilience is characterized by the degree of tolerance when it is subjected to targeted attack or random failure^33^. In present study, the resilience of the causal brain network was studied by removing nodes in order of decreasing node betweenness. We first selected the node with largest node betweenness and removed it from the network, and then recalculated the global efficiency of the remaining network. After this, we repeated this process incrementally remove the nodes until two nodes remained.

### Statistical analysis

A paired t-test was performed to measure differences of GC networks between stages. Before performing paired t-test, a z-score transformation was performed to make the sample fit the normal distribution (Eq. 1 in Table 4).

**Table 4:** Z-score transforming formula.

Name	Formula	Remarks
Z-score transforming	$$\begin{array}{c}z=\frac{GC-u}{\sigma }\,\end{array}$$Eq. 1	$GC$: causality connection values in causal connection matrix. $u$: the average of all values in the whole connection matrix. $\sigma$: the standard deviation of all values in the entire connection matrix.
